# The State of the Organs of the Female Reproductive System after a 5-Day “Dry” Immersion

**DOI:** 10.3390/ijms24044160

**Published:** 2023-02-19

**Authors:** Elena Yu. Gorbacheva, Konstantin A. Toniyan, Yulia A. Biriukova, Nadezhda A. Lukicheva, Oleg I. Orlov, Valery V. Boyarintsev, Irina V. Ogneva

**Affiliations:** 1 Cell Biophysics Laboratory, State Scientific Center of the Russian Federation Institute of Biomedical Problems of the Russian Academy of Sciences, 123007 Moscow, Russia; 2 Gynecology Department, FGBU KB1 (Volynskaya) UDP RF, 121352 Moscow, Russia; 3 Emergency and Extreme Medicine Department, FGBU DPO CGMA UDP RF, 121359 Moscow, Russia

**Keywords:** microgravity, ovary, endometrium, uterus, anti-Mullerian hormone, inhibin B, follicle-stimulating hormone, luteinizing hormone, progesterone

## Abstract

The impact of weightlessness on the female reproductive system remains poorly understood, although deep space exploration is impossible without the development of effective measures to protect women’s health. The purpose of this work was to study the effect of a 5-day “dry” immersion on the state of the reproductive system of female subjects. On the fourth day of the menstrual cycle after immersion, we observed an increase in inhibin B of 35% (*p* < 0.05) and a decrease in luteinizing hormone of 12% (*p* < 0.05) and progesterone of 52% (*p* < 0.05) compared with the same day before immersion. The size of the uterus and the thickness of the endometrium did not change. On the ninth day of the menstrual cycle after immersion, the average diameters of the antral follicles and the dominant follicle were, respectively, 14% and 22% (*p* < 0.05) higher than before. The duration of the menstrual cycle did not change. The obtained results may indicate that the stay in the 5-day “dry” immersion, on the one hand, can stimulate the growth of the dominant follicle, but, on the other hand, can cause functional insufficiency of the *corpus lutea*.

## 1. Introduction

Space exploration opens up new possibilities for the development of mankind. At the same time, maintaining the health of the various systems in the body during space flight and the subsequent adaptation to the Earth’s gravity continues to be an important task. In this context, little attention has been paid to the health of the reproductive system since a change in its condition does not pose an immediate critical threat to life.

Although space exploration has been historically dominated by men, more than 65 women have already made at least one space flight, and for them, the state of the reproductive system is of fundamental importance from at least two points of view. On the one hand, healthy aging in the female body depends on hormonal balance, which supports the health of the reproductive system. On the other hand, the average age of women on their first flight is 38 years [1], which, even under Earth conditions, is considered a late reproductive age. Despite the fact that the average age for the birth of the first child in women preparing for space flight is about 35 years [1], many of them postpone starting a family until they have made several space flights [2]. In addition, under the conditions of space flight, women often suppress the menstrual cycle by taking oral contraceptives [3]. After a space flight, some women are able to successfully conceive, even with the use of assisted reproductive technologies [4]. However, the proportion of unsuccessful reproductive efforts is quite high but the data are insufficient to assess the impact of space flight on reproductive function [2,4], as well as the state of the female reproductive system after space flight [1].

Research under space flight conditions is a priority; however, obtaining reliable results is difficult due to a small sample size. Therefore, various ground-based models are used, primarily the 6° head-down-tilt bed-rest model. This model has been used to study the state of the female reproductive system [5] but the data are scarce and contradictory. In one study, after a 17-day 6° head-down-tilt bed-rest test, there was no change in the length of the menstrual cycle [6]. In another study, the authors noted luteal phase deficiency in 3 out of 12 subjects by measuring progesterone levels [7]. However, despite the fact that female subjects participated in complex bed-rest experiments [8,9,10,11], nevertheless, the reproductive system was not the main priority.

Another generally accepted ground-based model is “dry” immersion [12,13,14]. However, similar to the bed-rest model, studies during “dry” immersion are most often aimed at the musculoskeletal, nervous, and cardiovascular systems [15,16,17,18]. Moreover, the first “dry” immersion study with women as the subjects that lasted 3 days was conducted in 2020 [19].

In this paper, we present the results of a study of the effect of 5-day “dry” immersion on the female reproductive system. The beginning of the exposure in the immersion bath was tied to the 10th day of the menstrual cycle and, accordingly, the exposure coincided with the late follicular phase and ovulation. We evaluated the content of the hormones involved in the maturation of follicles and the formation of *corpus lutea* and performed an ultrasound examination aimed at determining the structural parameters of the organs of the female reproductive system.

## 2. Results

### 2.1. The Levels of Hormones Involved in the Functioning of the Reproductive Tissue

**Figure 1:**
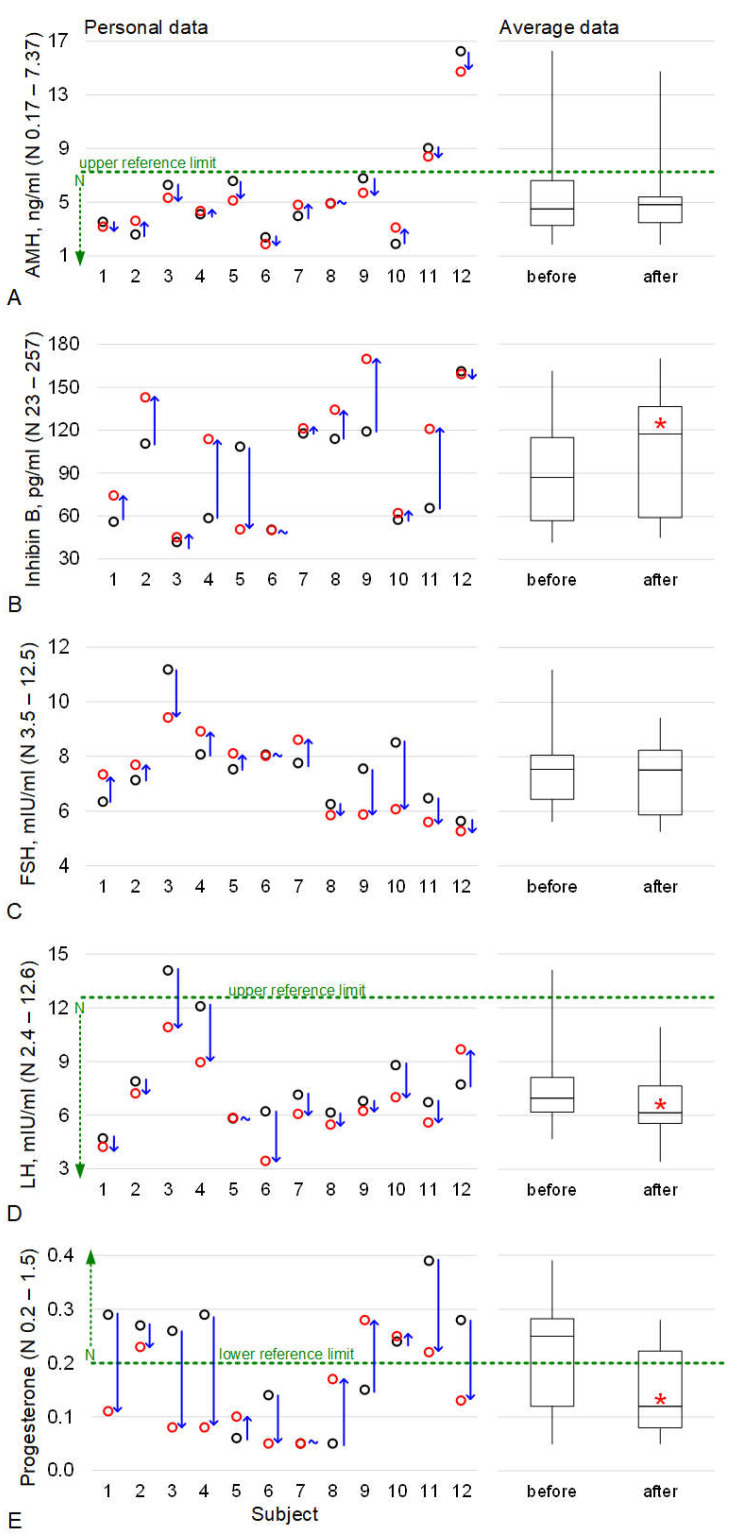
The concentration of hormones before and after a 5-day “dry” immersion. (**A**) anti-Mullerian hormone (AMH), (**B**) inhibin B, (**C**) follicle-stimulating hormone (FSH), (**D**) luteinizing hormone (LH), (**E**) progesterone (PG). The individual data are presented on the left side of each panel (1–12 are the conditional numbers of the subjects). The black circles indicate the values before immersion, the red circles indicate the values after immersion, and the direction of change is indicated in blue (↑—increase, ↓—decrease, ~—unchanged). Data are presented on the right side of each panel for the median ± range group. Before—value before immersion. After—value after immersion. * *p* < 0.05 in comparison to “before”. The green dotted lines indicate the upper or lower limit of the norm, if, for some subjects, the values were outside the reference range. The reference concentration is shown on a vertical scale for each hormone.

Anti-Mullerian hormone is a marker of ovarian reserve, in particular, the pool of primary follicles. The median value of anti-Mullerian hormone (AMH) concentrations in the blood serum before and after immersion did not differ significantly (Figure 1A). The individual data showed that the concentration of AMH before and after immersion did not go beyond the reference values, except for two subjects. In these subjects, the AMH concentrations before immersion were above the upper limit of normal; after immersion, these levels decreased but did not reach the normal level.

Like AMH, inhibin B is a marker of the ovarian reserve but it marks the pool of secondary, pre-, and antral follicles. After immersion, the concentration of inhibin B increased; the median value after immersion was 35% higher (*p* < 0.05) than before immersion (Figure 1B). The individual data showed that the concentration of inhibin B before and after immersion remained within the normal range in all subjects.

The concentration of follicle-stimulating hormone (FSH), on average, did not change after immersion; in all subjects, the values before and after immersion were within the normal range (Figure 1C).

In 10 out of 12 subjects, the concentration of luteinizing hormone (LH) decreased after immersion; in 1 it did not change and in 1 it increased (Figure 1D). The median value after immersion was 12% lower (*p* < 0.05) than before immersion. In one subject, the LH concentration exceeded the reference level before immersion; however, after immersion, it decreased and corresponded to the norm.

The concentration of progesterone (PG) generally decreased after immersion among the subjects and in 8 of 12 subjects, it fell below the lower limit of the norm (Figure 1E). However, in five subjects, even before immersion, the PG concentration was below the norm. The median value after immersion was lower than before immersion by 52% (*p* < 0.05).

### 2.2. The Data of the Ultrasound Examination of the Organs of the Reproductive System

**Figure 2:**
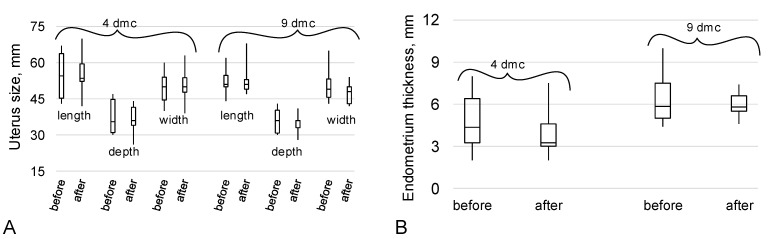
The size of the uterus (**A**) and the thickness of the endometrium (**B**) on the 4th and 9th days of the menstrual cycle (dmc) before and after a 5-day “dry” immersion.

According to ultrasound data, the size of the uterus before and after immersion did not differ on both the fourth and ninth days of the menstrual cycle (dmc) (Figure 2A). Endometrial thickness also did not change before and after immersion on the corresponding days of the menstrual cycle (Figure 2B).

**Figure 3:**
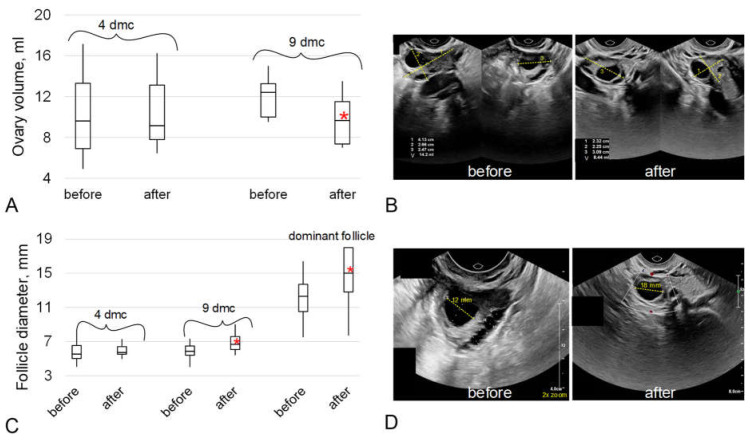
Ovarian volume and follicle diameter before and after a 5-day “dry” immersion on days 4 and 9 of the menstrual cycle. (**A**) Average ovarian volume for the group. (**B**) An example of an ultrasound picture: right ovary, 9th day of the menstrual cycle of the same subject before and after immersion—ovary volume decreased from 14 mL to 8.4 mL. Yellow dotted lines show the ovary sizes in the three dimensions, which are indicated by numbers 1, 2, 3 in the image (**C**) Average follicle diameter for the group. (**D**) An example of an ultrasound picture: 9th day of the menstrual cycle of the same subject before and after immersion—dominant follicle diameter increased from 12 mm to 18 mm. Yellow dotted lines mark the size of the dominant follicle (a 2x zoom is used in the “before” picture). * *p* < 0.05 in comparison to “before” of the same day of the menstrual cycle (dmc).

The average volume of the ovaries of the subjects on the fourth day of the menstrual cycle did not differ before and after the 5-day “dry” immersion. However, on the ninth day of the menstrual cycle, the ovarian volume after immersion decreased, on average, for the group; the median value after immersion was 22% lower (*p* < 0.05) than before immersion (Figure 3A,B).

The average diameter of the antral follicles on the fourth day of the menstrual cycle before and after immersion did not differ significantly, but on the ninth day of the cycle after immersion, it increased by 14% (*p* < 0.05), as did the diameter of the dominant follicle by 22% (*p* < 0.05).

## 3. Discussion

To date, there are practically no data on the impact of space flight or simulated weightlessness on the female reproductive system. The results of experiments on rodents are also scarce and incomparable due to the use of different species. Thus, in female Sprague–Dawley rats with a normal cycle, antiorthostatic suspension led to an elongation of the estrous cycle, which was characterized by more time spent in the diestrus stage and less time spent in the oestrus stage. In addition, antiorthostatic suspension was associated with a decrease in the serum estradiol concentration, the levels of LH and FSH in the serum and pituitary gland did not change, the level of progesterone in the serum did not change, and the weight of the ovaries and uterus did not change but the weight of the pituitary gland decreased [20]. However, Holets H.M. et al. showed that after space flight (shuttle missions STS-131, 133, 135), female mice experienced cycling arrest, loss of *corpus lutea*, and decreased estrogen receptor expression in the uterus [1]. In vitro experiments with mouse ovarian tissues showed that under conditions of simulated microgravity for 48 and 96 h, there was a decrease in the number of secondary follicles per unit area, there was no proliferation of granulosa cells, and there was no secretion of growth differentiation factor-9 (GDF9) by oocytes [21]. Rodent studies allow us to study the subtle mechanisms of the migration of primary germ cells and the formation of a pool of primordial follicles, as well as their recruitment to growth; however, in relation to humans, one should take into account the different ovulatory cycle (menstrual vs. estrus) and species-specific regulatory factors. 

In humans, as in other mammals, the formation of follicles begins during embryogenesis. Primordial germ cells simultaneously divide and migrate to the gonadal ridge where their cell cycle stops at the stage of the prophase of the first meiotic division, and a primordial follicle is formed with a diameter of less than 0.1 mm [22,23]. The maximum number of these follicles is observed around the 20th week of the development of the female fetus (more than 7 million), and then their death begins, which continues throughout the life of the woman [23,24]. These follicles form the ovarian reserve and a cohort of growing follicles are recruited from their pool. 

The activation of the growth of the primordial follicle leads to the surrounding squamous epithelial cells (pre-granulosa cells) becoming cubic granulosa cells and the formation of the primary follicle. The further growth of the oocyte, proliferation of granulosa cells up to 2 or more layers, and formation of the basement membrane and theca cells around it lead to the formation of a secondary follicle. The interaction between the oocyte and its surrounding cells determines the growth of the follicle [25]. The oocyte produces the GDF9 and BMP15 factors that stimulate the growth of granulosa cells [26]. Granulosa cells produce AMH, which, in turn, blocks the recruitment of new primordial follicles, activins that stimulate theca cell growth, and inhibins that block FSH secretion. Theca cells also produce growth factors TGFβ and LH receptors; however, activins block steroidogenesis in theca cells at this stage [25,27]. This preantral follicle is about 0.2 mm in size [28] and does not depend on gonadotropins.

The follicle continues to grow and a fluid-filled cavity (antrum) begins to form within it, dividing the granulosa cells into mural granulosa and cumulus cells, directly surrounding the oocyte. The small antral follicle is 0.2–0.4 mm in size [28] and at this stage, FSH receptors appear on granulosa cells. Follicle growth begins to depend on the hypothalamo–pituitary–gonadal axis [23,29]. From the pool of antral follicles, which are 2–5 mm in size [23,30], under the action of FSH and LH, the dominant follicle will mature and ovulate. Theca cells with receptors for LH under their control begin the synthesis of androstenedione from which estradiol is synthesized in granulosa cells under the control of FSH. Increasing concentrations of inhibin B and estradiol suppress FSH secretion and stimulate the formation of LH receptors on granulosa cells [25,31]. The peak release of LH leads to ovulation. The granulosa and theca cells remaining after ovulation are luteinized under the action of LH and begin to produce progesterone [25,27]. 

The cycle of follicle growth from primordial to ovulatory takes about 175 days [32], i.e., about 6–7 menstrual cycles. There is no single point of view on the growth dynamics of the recruited follicles [23]; however, taking into account the duration of the full cycle, we can say that the antral follicles we assessed after immersion were in the growth phase during immersion. 

The results obtained indicate that the AMH content did not differ before and after immersion (Figure 1A), which may indicate that the recruitment of the pool of dormant primordial follicles did not change. Notably, the possible effect of the 5-day immersion on the pool of primordial follicles can only be assessed after about six months, due to the duration of the follicle growth cycle (see above).

However, the content of inhibin B significantly increased after immersion (Figure 1B). Inhibin B is synthesized by the granulosa cells of the growing follicles so we can assume an increase in the number of cells and/or an increase in the expression of inhibin B. The latter seems more likely since the diameter of the follicle, according to ultrasound, did not change on this (fourth) day of the cycle before and after immersion (Figure 3C). Before the small antral follicle stage, both AMH and inhibin B are synthesized by the granulosa cells of the growing follicle [27,33] but we did not observe changes in the AMH content. The expression of inhibin B continues at the stage of a large antral follicle so it can be assumed that changes in granulosa cells take place at this stage.

The peak value of inhibin B content is observed at a follicle diameter of about 11 mm [31] and, like estradiol, inhibin B suppresses FSH synthesis in the mid-follicular phase [31]. We analyzed the FSH content on the fourth day of the cycle and found no change after immersion (Figure 1C). However, on the ninth day of the cycle after immersion, the diameter of the antral follicles was significantly higher, as well as that of the dominant follicle (Figure 3C). Moreover, the diameter of the dominant follicle was, on average, more than 15 mm, which may indirectly indicate a shortening of the follicular phase and approaching ovulation. However, it should be noted that the subjects did not notice a change in the length of the menstrual cycle during which they were in dry immersion conditions from the usual duration.

However, the level of LH in the blood on the fourth day of the cycle was significantly lower after immersion than before (Figure 1D). The decrease in LH may be associated with a decrease in its secretion by the pituitary gland but we did not find similar data in humans under microgravity conditions. Notably, in rodents after antiorthostatic suspension, a decrease in the mass of the pituitary gland was observed, although without a change in the content of gonadotropins [20]. On the other hand, a decrease in LH in the blood may be due to an increase in its utilization in the ovarian tissue. Inhibin B inhibits the action of activin, which suppresses the synthesis of LH receptors [25]. In other words, inhibin B has a stimulating effect on the expression of the LH receptor by theca cells, which, in turn, can lead to an increase in the uptake of LH from the blood and a decrease in its concentration there.

Luteinizing hormone leads to the formation of *corpus lutea* and controls its synthesis of progesterone. Accordingly, the decrease in LH could explain the decrease in blood progesterone observed after immersion (Figure 1E). Moreover, after bed rest, a decrease in progesterone was also noted in women [7] and the absence of *corpus lutea* formation in mice after space flight [1]. However, on the fourth day of the menstrual cycle, when we conducted the study, the *corpus lutea* of the previous cycle regressed and a new one will form only after ovulation.

However, progesterone synthesis begins even before ovulation LH surge by granulosa cells through the prostaglandin E2/EP2 receptor pathway stimulated by GDF9 [34,35,36]. If we rely on the above assumption about the shortening of the follicular phase and the shift of the ovulation time to the left, then it can be assumed that the decrease in progesterone content is associated with a possible decrease in GDF9 synthesis under conditions of simulated microgravity, which was shown in in vitro experiments with mouse tissues [21]. Although it should be noted that progesterone, in minimal amounts, is also synthesized in somatic tissues, which we have not studied, which makes our assumptions rather speculative.

**Figure 4:**
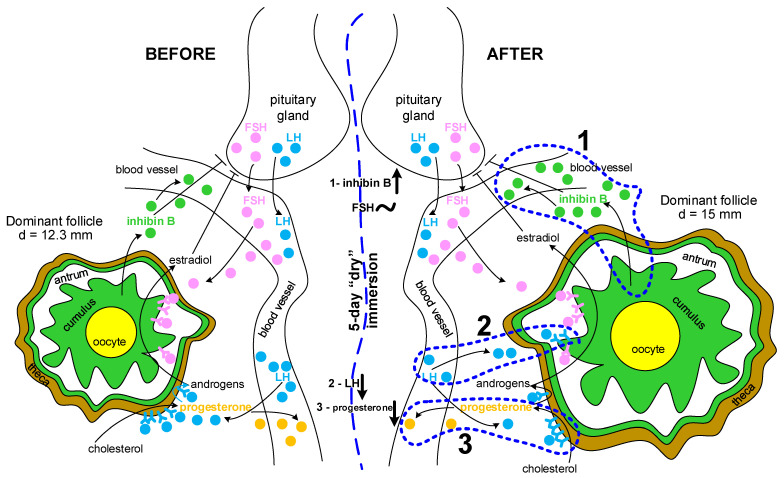
Possible scheme of events leading to a change in the concentration of hormones and the diameter of the dominant follicle after a 5-day “dry” immersion. Changes in hormone content are labeled as follows: ↑—increase, ↓—decrease, ~—unchanged. (1) As a result of staying in “dry” immersion from 10 to 15 days during the previous menstrual cycle, an increase in the number of granulosa cells of the growing antral follicles occurs. This leads to an increase in the growth of the dominant follicle in the next cycle and an acceleration of its maturation. Thus, the diameter of the dominant follicle increases, and, due to the increase in the number of granulosa cells, the production of inhibin B increases (marked with a small green circle), as well as its concentration in the blood. (2) Accelerated maturation of the follicle leads to an earlier appearance of LH receptors on granulosa cells and, consequently, to their greater utilization in the bloodstream. Accordingly, the concentration of LH (marked with a small light-blue circle) in the blood falls. (3) A decrease in the concentration of LH in the blood leads to its deficiency for receptors located on the theca cells and, consequently, to a decrease in the synthesis of progesterone (marked with a small yellow circle) and a decrease in its concentration in the blood. The arrows indicate the migration of hormones, the blue dashed line indicates the fluxes that have changed after immersion.

Summarizing the results obtained and the above assumptions, we can propose a possible sequence of events (Figure 4) that lead to an increase in the diameter of the dominant follicle, an increase in the concentration of inhibin B in the blood, and a decrease in the concentration of LH and progesterone. 

## 4. Materials and Methods

### 4.1. Experimental Design

Sixteen healthy women were recruited for the experiment. For the study of the reproductive system described in this article, 12 out of 16 subjects were selected and 4 subjects were excluded because they were taking oral contraceptives. Of the 12 subjects, 6 received a lactoferrin preparation once a day from days 1 to 29 of the experiment and the remaining 6 received a placebo. In our study data, there was no difference between the parameters of subjects who received the drug and those who received a placebo. Therefore, we consider the general group to be the 12 selected subjects. 

**Figure 5:**
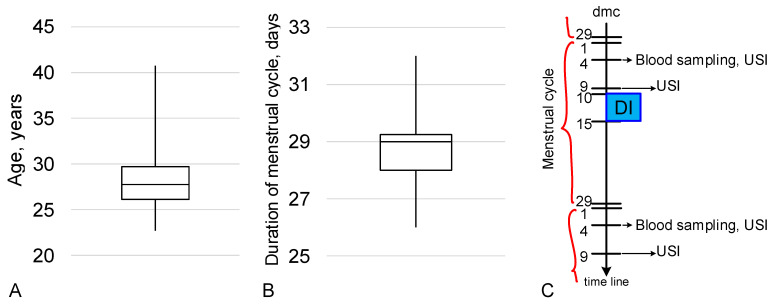
Characteristics of the group of subjects. (**A**) Average age of the subjects. (**B**) Duration of the menstrual cycle. (**C**) Cyclogram of the study of the state of the reproductive system. dmc—day of menstrual cycle, DI—“dry” immersion, USI—ultrasound imaging. The cyclogram is given for the average duration of the menstrual cycle in the group of subjects (29 days) and the start of exposure at 10 dmc.

The age of the subjects ranged from 22.7 to 40.8 years and the average age was 27.8 years (Figure 5A). The duration of the menstrual cycle during the experiment ranged from 26 to 32 days, with an average of 29 days, and the subjects did not notice a change in the lengths of their menstrual cycles (Figure 5B).

Exposure under the conditions of “dry” immersion for 5 days was carried out exactly as described earlier for a 3-day immersion [37]. It is known that most physiological systems under the conditions of a “dry” immersion respond after 3 days, and the maximum acute changes are observed after 5–7 days [14]. Since the ongoing immersion study is a complex experiment aimed at studying all the physiological systems of the body, a duration of 5 days was chosen. The exposure was conducted at the “dry” immersion facility of the Institute of Biomedical Problems, Russian Academy of Science [14,19]. Subjects, wearing a shirt and trunks, were placed on waterproof fabric and immersed in a deep bath (the water temperature in the bath was maintained at 32.5 ± 2 °C to avoid body cooling through the waterproof fabric) up to neck level in a supine position. The area of the fabric’s surface considerably exceeded that of the water’s surface. The high elasticity properties of the fabric artificially created conditions similar to zero gravity via floatation. The folds of the waterproof fabric allowed the person’s body to be freely enveloped on all sides and avoid any contact of the body with water. Subjects lay in the immersion bath without any physical activities. The average time spend outside the immersion bath (for hygiene procedures and certain experimental examinations) did not exceed 30 min per day. The crew, consisting of a doctor, an assistant, and a technician, provided 24 h monitoring of participants’ health and the working conditions of the technical equipment [37]. 

The beginning of exposure under the conditions of “dry” immersion (DI) in 7 subjects was on the 10th day of the menstrual cycle (dmc), in 3 subjects at 12 dmc, in 1 subject at 13 dmc, in 1 subject at 15 dmc. All subjects at 4 dmc underwent blood sampling (strictly on an empty stomach) for estimating the levels of hormones and an ultrasound examination for estimating the antral follicles. At 9 dmc, only an ultrasound examination was performed for estimating the dominant follicle. The study was carried out before and after exposure to dry immersion conditions (Figure 5C).

For our investigation of the reproductive system, all subjects were treated at Gynecology Department Clinical Hospital # 1 (Volynskaya, Moscow, Russia). All subjects were in a stable clinical condition with no clinical, microbiological, or laboratory evidence of infection, encephalopathy, renal failure, or comorbidities including heart failure, pulmonary disease, malignancy, or diabetes mellitus. Written informed consent was obtained from each subject prior to participation in the study. The study design and procedures were approved by the Biomedicine Ethics Committee of the Institute of Biomedical Problems, Russian Academy of Sciences (Physiology Section of the Russian Bioethics Committee, Russian Federation National Commission for UNESCO, Permit #615/MSK/06/06/22) and conformed to the Declaration of Helsinki.

### 4.2. Blood Sampling and Measurements of Hormones

Blood samples were collected at around 8.30 am for all subjects at 4 dmc before and after DI. Serum was separated by centrifugation and measurements were made immediately without freezing. Follicle-stimulating hormone (FSH), luteinizing hormone (LH), anti-Mullerian hormone (AMH), and progesterone (PG) were determined by electrochemiluminescence immunoassay (Elecsys^®^ reagents and Cobas e411 analyzer, Roche Diagnostics Ltd., Rotkreuz, Switzerland). Inhibin B was measured using an enzyme-linked immunosorbent kit (#A81303, Beckman Coulter, Inc., Brea, CA, USA) by an automatic analyzer (Personal LAB, Adaltis S.r.l., Rome, Italy).

### 4.3. Ultrasound Examination

Ultrasound examinations of the organs of the reproductive system were performed at 4 dmc and 9 dmc before and after DI using an EPIQ7 ultrasound system with a broadband curved array transducer with PureWave crystal technology C10-3v (Philips, Amsterdam, Netherlands). Imagery for determining the size of the uterus and ovaries, the diameter of the follicles, as well as counting their number, was performed using the nSIGHT integrated imaging system (Philips, Amsterdam, Netherlands).

### 4.4. Statistical Analysis

We used non-parametric statistics due to the small number of subjects, high variability of the levels of the baseline parameters, and non-normal distribution. To compare “before” and “after”, Wilcoxon tests were used for post hoc comparisons with statistical significance at the *p* < 0.05 level. Data were presented as median ± range.

## 5. Conclusions

Summarizing the above, we can conclude that even a 5-day stay under the conditions of “dry” immersion in the late follicular phase/ovulation affects the state of the female reproductive system. The ovarian reserve remains intact since its main marker—anti-Mullerian hormone—does not change. The growth of antral follicles is likely stimulated—their diameter and that of the dominant follicle increase, as well as the concentration of inhibin B. At the same time, the concentrations of luteinizing hormone and progesterone on the fourth day of the cycle after immersion were significantly reduced compared to the corresponding values before immersion. Very cautiously, we can assume that this was due to a shortening of the follicular phase and a shift of ovulation to the left, although other explanations are possible, which would require further research. 

## 6. Limitations of the Study

There are several limitations of this study:

- the small number of subjects and high variability of the baseline parameters;
- the phase of the menstrual cycle was determined by the date of the beginning of the last menstrual period but, due to the different reproductive histories, ages, etc., it could have shifted the longevity of the phases;
- due to technical limitations during the organization of the experiment, the different days of the menstrual cycle at the beginning of exposure in the immersion bath could have caused the variability of some of the effects;
- due to the same reasons mentioned above, our examinations were possible only on the fourth and ninth days of the menstrual cycle. The optimal dmc for estimating hormone levels is 2–3 dmc, for counting antral follicles it is 5–6 dmc, and for estimating the dominant follicle it is 11–12 dmc. In addition, it would be better to expand the range of detectable hormones involved in the functioning of the reproductive tissue, as well as the points of measurement. The lack of subject follow-up over future menstrual cycles and, as the result, the lack of data on the dynamics of the measured parameters is one of the most significant limitations.

## Data Availability

All data generated or analyzed during this study are included in this article.
